# Halogen bonds, chalcogen bonds, pnictogen bonds, tetrel bonds and other σ-hole inter­actions: a snapshot of current progress

**DOI:** 10.1107/S2053229623004072

**Published:** 2023-05-22

**Authors:** Lee Brammer, Anssi Peuronen, Thomas M. Roseveare

**Affiliations:** a Department of Chemistry, University of Sheffield, Sheffield, S3 7HF, United Kingdom; b Department of Chemistry, University of Turku, FI-20014 Turku, Finland; The University of Melbourne, Australia

**Keywords:** halo­gen bond, chalcogen bond, pnictogen bond, tetrel bond, sigma-hole, noncovalent inter­action, supramolecular chemistry

## Abstract

We report here on the status of research on halo­gen bonds and other σ-hole inter­actions involving *p*-block elements in Lewis acidic roles, such as chalcogen bonds, pnictogen bonds and tetrel bonds. A brief overview of the available literature in this area is provided *via* a survey of the many review articles that address this field. Our focus has been to collect together most review articles published since 2013 to provide an easy entry into the extensive literature in this area. A snapshot of *current* research in the area is provided by an introduction to the virtual special issue com­piled in this journal, com­prising 11 articles and entitled ‘*Halogen, chalcogen, pnictogen and tetrel bonds: structural chemistry and beyond*.’

## Abstract 2

A com­pilation of review articles on σ-hole inter­actions published since 2013 is presented alongside an overview of the 11 articles in the special issue on this topic.

## Introduction

Halogen bonding and related inter­molecular (and sometimes intra­molecular) inter­actions in which *p*-block elements in groups other than Group 17 serve in a Lewis acidic role have been and continue to be extensively investigated. Their ap­plica­bility in supra­molecular assembly is similarly well studied, with numerous reports of applications in crystal en­gin­eering, mol­ecular recognition, catalysis, polymers and soft matter, materials chemistry, structural biology and medicinal chemistry. Many of these areas are the subjects of reviews (see Section 2 for more details).

It has been noted that examples of this family of inter­actions, particularly halo­gen bonds, are implicit in reports of com­pounds and observed behaviour as far back as the early 19th century (Colin & Gaultier de Claubry, 1814 ▸; Colin, 1814 ▸; also see the note in Section 5). Extensive historical perspectives can be found in several substantial review articles (Cavallo *et al.*, 2016 ▸; Gilday *et al.*, 2015 ▸). Definition, identification and understanding of such inter­actions evolved slowly until the mid-20th century when work by Mulliken and others classified inter­actions of I_2_ with Lewis basic solvents as electron donor–acceptor com­plexes (Benesi & Hildebrand, 1948 ▸; Mulliken, 1950 ▸) based on UV–Vis spectroscopic studies. Com­plementary crystallographic studies of dihalo­gen inter­actions with such solvents by Hassel and co-workers then revealed the now well-known linear geometry and short inter­action distances (Hassel & Hvoslef, 1954 ▸). This class of inter­actions became well established in the 1960s and 1970s, as reviews by Bent on donor–acceptor inter­actions (Bent, 1968 ▸) and by Alcock on *secondary bonding* (Alcock, 1972 ▸) attest. The prevailing description at that time was of an electron donor–acceptor inter­action, with the Lewis acidic *p*-block atom involved in the electron-acceptor com­ponent. This bonding description is still common in current studies, but is dominant for the strongest inter­actions, whereas an electrostatic model for the inter­actions first advanced 15 years ago, and usually referred to as the σ-hole model (Clark *et al.*, 2007 ▸; Politzer *et al.*, 2013 ▸), is generally thought to provide the best description of weak to moderate strength inter­actions. Indeed, the electrostatic description has become sufficiently prevalent that this class of inter­actions is often referred to as σ-hole inter­actions, as we have done in the title of this article, although the term perhaps lacks universality since it implies a universal (electrostatic) bonding model. Alcock’s earlier term *secondary bonding* is not prescriptive, but refers to inter­actions other than primary (covalent) bonds. Subclasses of inter­actions involving elements from a particular *p*-block group in the Lewis acidic role are most commonly named after the class of elements that com­prise the group, the most common being halo­gen bonds (Group 17), chalcogen bonds (Group 16), pnictogen bonds (Group 15) and tetrel bonds (Group 14). In a tutorial review entitled ‘*Hypervalency, secondary bonding and hydro­gen bonding: siblings under the skin*,’ which also benefits from some historical perspective, Crabtree (2017 ▸) provides an important reminder that there is a continuum of behaviour, both in geometry and bonding des­cription, from weak secondary bonding through to the strongest inter­actions that evolve into hypervalency of the main group elements. This continuum description applies equally to hydro­gen bonding.

**Figure 1:**
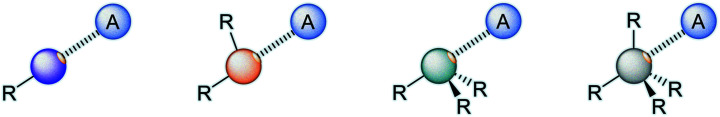
Left to right: halo­gen bond, chalcogen bond, pnictogen bond and tetrel bond. Each *p*-block atom is shown with its most common number of substituents (*R*) engaging in an inter­molecular (σ-hole) inter­action with a Lewis basic acceptor group (*A*).

After a period of relative dormancy, the field of halo­gen bonding has grown enormously in the past 25 years, accelerated initially by studies in the late 1990s/early 2000s that made clear the similarities between halo­gen bonding and the more widely studied hydro­gen bonding, notably the matrix-isolation and gas-phase rotational spectroscopy studies of Legon and co-workers (Legon, 1999 ▸), and the introduction by Resnati, Metrangolo and colleagues of perfluoro­alkyl and -aryl halides to enable strong directional halo­gen bonding in the condensed phases (Corradi *et al.*, 2000 ▸). The evolution of halo­gen bonding is such that not only are there many reviews on this topic (*e.g.* Metrangolo *et al.*, 2005 ▸; Rissanen, 2008 ▸; Brammer *et al.*, 2008 ▸; Fourmigué, 2009 ▸; Cavallo *et al.*, 2010 ▸; Bertani *et al.*, 2010 ▸; Parisini *et al.*, 2011 ▸; Erdélyi, 2012 ▸), but many reviews even focus on specific applications of halo­gen bonding (Section 2.1). Chalcogen bonds are the next most widely studied class of secondary bonding inter­actions, their understanding and application having advanced considerably in the past decade. Reviews that focus specifically on these or the other classes of inter­actions are now also plentiful. Thus, we have endeavoured to bring together a com­pilation of the many topics that have been reviewed in the sections below, along with tabulated and categorized lists of reviews [including survey articles in which the Cambridge Structural Database (CSD; Groom *et al.*, 2016 ▸) has been used as the primary source of experimental data to identify inter­actions, and reviews that include new com­putational studies of the com­pounds being surveyed]. The aim has been to provide an ease of departure into the extensive literature on the classes of inter­actions (Fig. 1 ▸) that are brought together in this special issue of *Acta Crystallographica Section C: Structural Chemistry*.

## Signposting reviews on secondary bonding: halo­gen bonds, chalcogen bonds, pnictogen bonds, tetrel bonds, *etc*.

In the following sections, we have very briefly summarized the broad areas covered by reviews on each class of secondary bonding inter­actions. Although a com­prehensive list would be desirable, accom­plishing this with certainty is difficult to ensure. Given the vast number of reviews, we have focused on those published in the past decade (2013–present). There are, of course, several key reviews that precede this period. A selection of these is noted in the *Introduction* (Section 1). There will inevitably be reviews published that are not listed in Tables 1 ▸–5 ▸ ▸ ▸ ▸; their omission is unintentional. There are also a number of collections of articles in journals that have been com­piled around this topic. A good example is the series of articles associated with the 203rd Faraday Discussion meeting on ‘*Halogen Bonding in Supra­molecular and Solid State Chemistry*,’ held in Ottawa, Canada, on 10–12th July, 2017 (see, for example, Clark, 2017 ▸; Brammer, 2017 ▸), which includes transcripts of the extensive discussion arising from the presented articles (Aakeröy *et al.*, 2017*a* ▸,*b* ▸,*c* ▸,*d* ▸). Section 3 (*vide infra*) provides an overview of the collection of articles in the special issue of this journal, with which the present article is associated.

### Reviews of halo­gen bonding

As noted in the *Introduction* (Section 1), halo­gen bonding was the first of the σ-hole inter­actions to receive renewed attention in the revival and blossoming of research over the past 25-plus years; it remains the most widely studied of these related inter­actions. This has led to a large number of reviews within the past ten years (Table 1 ▸) and has even resulted in the publication of a number of books focusing on halo­gen bonding (Cavallo *et al.*, 2015 ▸; Kolář *et al.*, 2015 ▸).

Reviews cover halo­gen bonding in the solid state, in solution and in the gas phase. A simple overview of the developing trends within these reviews reveals that, within the past decade, the focus has shifted from the general reviews that dominated the first half of the decade to reviews focusing on furthering the understanding of halo­gen bonding, *e.g.* utilizing com­putational input, with reviews centred on specific applications of halo­gen bonding becoming more prominent in the latter half of the past decade. This snapshot of the field illustrates the promise for the use of halo­gen bonding in a variety of applications and fields, including materials chemistry, organo­catalysis and biologically/medically relevant areas. These trends towards employing halo­gen bonding to modify the properties of materials or the reactivity/activity of mol­ecules are echoed in the reviews of the related inter­actions, alluding to the role that σ-hole inter­actions will play in future scientific research. Lists of reviews on chalcogen bonding, pnictogen bonding and tetrel bonding are provided in Tables 2 ▸–5 ▸ ▸ ▸.

### Reviews of chalcogen bonding

In this section, a collection of reviews that are related specifically to chalcogen bonding is provided. A list of these reviews can be found in Table 2 ▸. Although these reviews focus primarily on the supra­molecular and crystal engineering aspects of the chalcogen bond, areas such as drug development and biological aspects, as well as materials chemistry, are also prevalent. Furthermore, the role of chalcogen bonding in organic synthesis and catalysis is covered in several of the listed reviews. A reader unfamiliar with the topic may find the overview of chalcogen bonding by Huber and co-workers a good starting point (Vogel *et al.*, 2019 ▸), while a general definition of the chalcogen bond has been provided through the IUPAC (Inter­national Union of Pure and Applied Chemistry) nomenclature process (Aakeröy *et al.*, 2019 ▸).

### Reviews of pnictogen bonding

The reviews listed in Table 3 ▸ are those dedicated exclusively to pnictogen (also pnicogen) bonding and cover this particular σ-hole inter­action mainly from a crystal engineering point of view, but also review the fundamental aspects of the pnictogen bond by summarizing articles that involve relevant com­putational results. To highlight some of these reviews, Varad­waj and co-workers provide four separate reviews (Varad­waj *et al.*, 2022*b* ▸,*c* ▸,*d* ▸,*e* ▸, 2023*b* ▸), each devoted to a specific atom of the pnictogen family and its respective pnictogen-bonded systems, based on crystal structures present in the Cambridge Structural Database (CSD). Furthermore, a CSD survey of coordination and organometallic com­pounds which involve pnictogen-bond motifs has been conducted by Mahmudov *et al.* (2022 ▸), while the review by Scheiner (2013*b* ▸) provides a com­prehensive look at the fun­damental aspects of the pnictogen bond. The significance of pnictogen bonding in areas such as catalysis and biology are highlighted in reviews listed in Table 5 ▸ that focus on multiple types of σ-hole inter­actions.

### Reviews of tetrel bonding

Similar to pnictogen bonding, the reviews of tetrel bonding listed in Table 4 ▸ concentrate largely in surveying crystallo­graphic data using the CSD, while some of these reviews pro­vide a com­prehensive look at the characteristic features of the tetrel bond by drawing conclusions mainly from com­putational studies or in conjunction with experimental data. Although not a review and therefore not listed in Table 4 ▸, a recent article by Varadwaj *et al.* (2023*a* ▸) is worth mentioning here since it proposes a definition of the tetrel bond. Some of the listed surveys look mainly at C atoms as the Lewis acidic centre, although reviews focusing on tetrel bonding involving its heavier congeners germanium, tin and especially lead are also listed. We note that the review by Laplaza *et al.* (2021 ▸) is placed in Table 4 ▸ rather than Table 5 ▸ since the authors place tetrel bonds in particular focus while analysing ‘new bonding situations’ using the highlighted com­putational method (Laplaza *et al.*, 2021 ▸).

### Reviews that include multiple σ-hole inter­action types

Associating each review strictly with a single class of inter­action (*i.e.* halo­gen, chalcogen, pnictogen or tetrel bond­ing) is not always possible and therefore in Table 5 ▸ we provide an extended list of reviews in which two or more of these σ-hole inter­actions are reviewed. This list also contains reviews that com­pare the features of tetrel, pnictogen, chalcogen and/or halo­gen bonding with hydro­gen bonds. Additionally, some of these reviews provide further reading on other types of noncovalent inter­actions beyond the σ-hole inter­actions introduced above, including aerogen (noble gas) bonding and triel (Group 13) bonding, as well as so-called π-hole inter­actions.

### Other σ-hole or related inter­actions

Although this review and the accom­panying special issue of the journal have focused on σ-hole inter­actions involving Group 14–17 elements in the Lewis acidic role, it has long been recognized that elements from other groups can also form analogous inter­actions. In most cases, these inter­actions are currently the subject of insufficient studies to warrant a review article. The involvement of noble gases in such secondary bonding inter­actions was reviewed by Alcock 50 years ago (Alcock, 1972 ▸). More recent work, under the classification of aerogen bonds has come from individual studies (*e.g.* Bauzá & Frontera, 2015 ▸). Inter­actions involving Group 13 elements as Lewis acids have been reviewed recently under the classification of triel bonds (Grabowski, 2020*b* ▸). Lewis acidic behaviour, however, is already well established, *i.e.* typical behaviour, of Group 13 elements. The description of triel bonding for these elements relies on describing the well-established chemistry in terms of a π-hole inter­action at the triel element. Analogies between the σ-hole description of inter­actions of Group 14–18 elements have also recently been evoked for weak inter­actions of Lewis bases with some transition-metal com­plexes of Groups 7, 8, 11 and 12 (Daolio *et al.*, 2021*a* ▸,*b* ▸; Legon & Walker, 2018 ▸; Bauzá *et al.*, 2020 ▸; Banerjee *et al.*, 2022 ▸; Gomila & Frontera, 2022 ▸). Caution should perhaps be observed here so as not to reimagine all of *d*-block coordination chemistry in terms of σ-holes, but some structural and bonding analogies between weak inter­actions involving these *d*-block elements and those involving *p*-block elements is clearly of value. A survey of related inter­actions from across the *s*- and *p*-blocks, and part of the *d*-block has been reported by Alkorta *et al.* (2020 ▸).

## The virtual special issue

The virtual special issue of this journal, which runs under the heading ‘*Halogen, chalcogen, pnictogen and tetrel bonds: structural chemistry and beyond*’ was conceived as providing a snapshot of current research activity and includes 11 articles that cover a variety of aspects associated with this class of inter­actions. There are articles that focus on structure, bond­ing and bond strength, articles that investigate co-operation or com­petition between different classes of inter­actions, articles that explore the relationship between these inter­actions and chemical or physical properties of the com­pounds and materials involved, and, although all articles involve characterization by single-crystal X-ray diffraction, a number have a prominent focus on other experimental techniques or are combined with inter­pretation from theoretical calculations. Halogen bonding and chalcogen bonding dominate these articles, consistent with the wider literature (*vide supra*).

**Figure 2:**
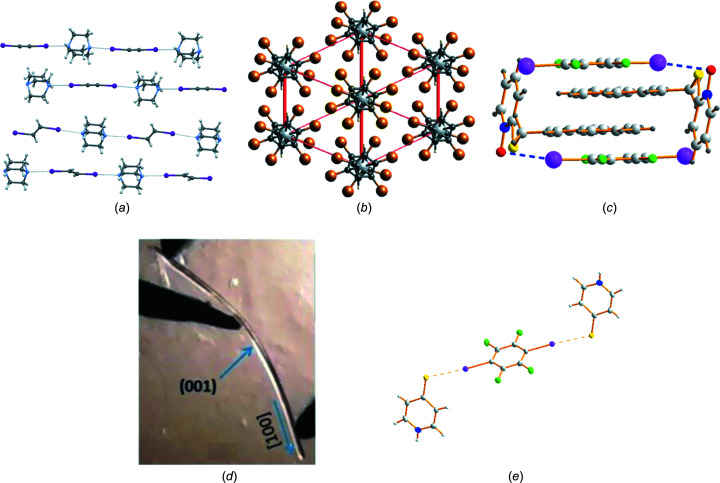
(*a*) C—I⋯N halo­gen bonding in the series C_2_I_2_·DABCO, C_2_H_2_I_2_·DABCO and C_2_H_4_I_2_·DABCO (Torubaev & Skabitskiy, 2022 ▸). (*b*) Polybromo­ferrocenes – multiple halo­gen-containing inter­actions (Blockhaus & Sünkel, 2022 ▸). (*c*) C—I⋯O halo­gen bonding and π-stacking in flexible NPTO (Wang *et al.*, 2023 ▸). (*d*) Bending crystals of 4-halobenzo­nitrile containing C—*X*⋯N≡C halo­gen bonds (Veluthaparambath *et al.*, 2022 ▸). (*e*) C—I⋯S halo­gen-bonded cocrystals – links to the nucleophilic substitution reaction (Mosquera *et al.*, 2023 ▸). All figures are reproduced from the cited references with permission.

Five articles focus on different aspects of the structure, bonding and resulting properties of halo­gen-bonded crystals (Fig. 2 ▸). Torubaev & Skabitskiy (2022 ▸; see also Perkins *et al.*, 2012 ▸) use X-ray crystallography to study the effect on C—I⋯N halo­gen bonds of the hydridization at the C atom across the series C_2_I_2_·DABCO, C_2_H_2_I_2_·DABCO and C_2_H_4_I_2_·DABCO (DABCO is 1,4-di­aza­bicyclo­[2.2.2]octa­ne), demonstrating that, in these linear halo­gen-bonded assemblies, halo­gen-bond lengths (I⋯N) follow the trend C(*sp*)—I⋯N < C(*sp* ^2^)—I⋯N < C(*sp* ^3^)—I⋯N. The crystallographic results are supported by calculations of electrostatic potentials. Blockhaus & Sünkel (2022 ▸) report the synthesis of a series of highly brominated ferrocenes [C_10_H_10–*n*_Br_*n*_Fe], with *n* = 4–9. The crystal structures of some of these com­pounds are de­scribed and exhibit C—H⋯Br hydro­gen bonds, C—Br⋯Br—C inter­actions, some of which are halo­gen bonds, and C—Br⋯π halo­gen bonds. These com­pounds are discussed in the broader context of other polybromo­ferrocenes and other polyhalo­ferrocenes, and analysed using Hirshfeld surface representations and by inter­molecular energy calculations. Wang, Wu and Jin report halo­gen-bonded cocrystals of di- and tri­iodo­per­fluoro­benzenes with a flexible thio­ether containing a strong halo­gen-bond acceptor pyridyl *N*-oxide group (Wang *et al.*, 2023 ▸). Accommodation of the halo­gen-bond donor species in the cocrystals involves a change in conformation of the flex­ible NPTO mol­ecule (NPTO is 2-{[(naphthalen-2-yl)meth­yl]sulfan­yl}pyridine 1-oxide) upon formation of C—I⋯O halo­gen bonds and π-stacking inter­actions between the electron-rich naphthyl groups of the NPTO mol­ecule and the electron-poor iodo­perfluoro­benzenes, which are inter­preted in terms of a π-hole description. The crystallographic studies are com­plemented by quantum chemical calculations. Saha and co-workers investigate the relationship between physical properties and halo­gen-bond strength. Specifically, they have examined the propensity for single crystals of 4-halo­ben­zenes to bend (Veluthaparambath *et al.*, 2022 ▸). In their article, they correlate the crystal structure of 4-iodo­benzo­nitrile and its brittle behaviour, which contrasts with the chloro and bromo analogues that exhibit elastic bending and plastic bending, respectively. The study is supported by density functional theory (DFT) calculations and a statistical analysis of C—*X*⋯N≡C halo­gen-bond geometries using the CSD. Finally, Mosquera *et al.* (2023 ▸) link halo­gen bonding and reactivity in their study of pyridine-4-thiol (4-mercapto­pyri­dine). Cocrystallization with the ditopic halo­gen-bond donor 1,4-di­iodo­tetra­fluoro­benzene leads to tautomerization of the thiol to give the zwitterionic analogue with thiol­ate and pyridinium groups. This zwitterion employs the sulfur as the halo­gen-bond acceptor (C—I⋯S, *R*_IS_ = 0.84) in the formation of a 2:1 cocrystal (C_5_H_5_NS·C_6_F_4_I_2_), in which these trimolecular supermolecules are further linked *via* N—H⋯S hydro­gen bonds. When the cocrystallization is pursued on a larger scale, with stirring, the solution containing the two cocrystal formers leads to a nucleophilic substitution reaction that results in the chloro groups of the CH_2_Cl_2_ solvent being replaced by the formation of new C—S bonds to give the zwitterionic form of 4-mercapto­pyridine. The resulting [CH_2_(SC_4_H_5_N)_2_]^2+^ dication crystallizes as its dichloride salt and exhibits C—S⋯Cl chalcogen bonds. Stabilization of the zwitterionic form of 4-mercapto­pyridine in solution by halo­gen bonding to C_6_F_4_I_2_ is suggested as enabling the sulfur to serve as a better nucleophile in its reaction with CH_2_Cl_2_.

**Figure 3:**
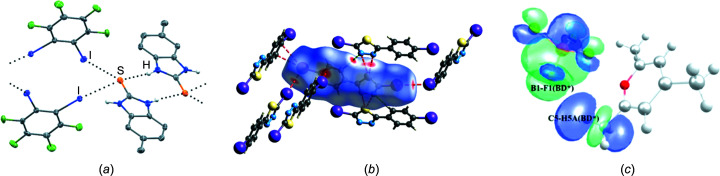
(*a*) C—I⋯S halo­gen bonding in organoiodine cocrystals of heterocyclic thio­nes (Watts *et al.*, 2022 ▸). (*b*) Competition between halo­gen and chalcogen bonds in halo­gen-bearing chalcogena­diazo­les (De Silva *et al.*, 2022 ▸). (*c*) B—F⋯O and B—F⋯C inter­actions and orbital analysis in 2,4,6-tri­methyl­pyrylium tetra­fluoro­borate (Mandal *et al.*, 2022 ▸). All figures are reproduced from the cited references with permission.

Three articles focus on the com­petition or co-operation of halo­gen bonds with other σ-hole class (secondary bonding) inter­actions (Fig. 3 ▸). Pennington and co-workers report a series of 18 cocrystals between heterocyclic thio­nes based on benzimidazole, benzoxazole or benzo­thia­zole with a variety of iodo­perfluoro­benzenes or tetra­iodo­ethene (Watts *et al.*, 2022 ▸), leading to structures that are rich in directional inter­molecular inter­actions. Persistent N—H⋯S hydro­gen bonding leads to two-dimensional (2D) tape motifs or dimers depending on the number of N—H groups available. These units are then further linked by C—I⋯S and/or C—I⋯I halo­gen bonds and occasionally by C=S⋯I—C chalcogen bonds. Aakeröy and co-workers have designed a library of mol­ecules based upon 1,3,4-chalcogena­diazo­les that carry a halo­gen substituent at the 2-position of the five-memberered ring and a 4-halophenyl group attached at the 5-position of the same ring (De Silva *et al.*, 2022 ▸). This family of mol­ecules contains two different halo­gen-bond donor groups, the strengths of which differ and are tuneable, as indicated by electrostatic potential calculations of their associated σ-holes. The five-membered ring provides a chalcogen-bond donor site and acceptor sites for both halo­gen and chalcogen bonds at the ring N atoms. The mol­ecules are linked into 2D assemblies, propagated along the *c* axis by halo­gen bonds (C—I⋯I, C—Br⋯Br and C—Br⋯I) and along the *a* axis *via* bifurcated chalcogen bonds involving both ring N atoms in the acceptor role. This outcome contrasts with a prediction based simply on σ-hole electrostatic potentials, which would suggest that the strongest inter­action would be halo­gen bonding to the ring N atom (position-3). The analysis of the inter­actions is supported by Hirshfeld surfaces, fingerprint plots and energy framework calculations (Spack­man & Jayatilaka, 2009 ▸; Spackman *et al.*, 2021 ▸), the latter indicating that the observed chalcogen bonds make a larger electrostatic contribution to the lattice energy than the C—*X*⋯*X* halo­gen bonds. Chopra, Hathwar and co-workers report the structure of the organic salt 2,4,6-tri­methyl­pyrylium tetra­fluoro­borate, C_5_H_2_Me_3_O^+^·BF_4_^−^, and present an extensive com­putational analysis of the short inter­molecular con­tacts to provide a description of the inter­action type (Mandal *et al.*, 2022 ▸). Among the inter­actions indicated by the topological analysis of the calculated electron density and accom­panying distributed atomic polarizability calculations are B—F⋯O inter­actions. Although not clearly described as either halo­gen or chalcogen bonds, NBO (natural bond orbital) analysis indicates inter­action of filled F(lone pair) with O—C(π*) orbitals. B—F⋯C inter­actions involving the *ortho*-methyl groups of the pyrylium ring and involving *ortho* ring C atoms are described as tetrel bonds.

**Figure 4:**
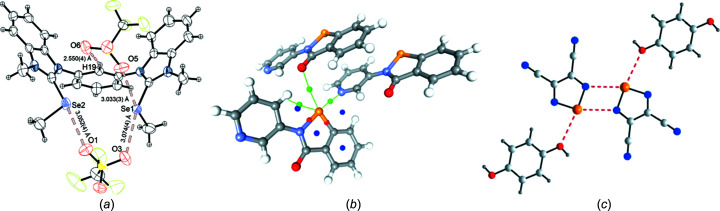
(*a*) C—Se⋯O chalcogen bonding in 1,3-bis­(benzimidazolium­yl)benzene-based chalcogen-bonding catalysts (Steinke *et al.*, 2023 ▸). (*b*) C—Se⋯N and C—Se⋯O chalcogen bonding in ebselen analogues, shown with critical points in electron density (Xu *et al.*, 2023 ▸). (*c*) N—Ch⋯N and N—Ch⋯O chalcogen bonding in 3,4-di­cyano-1,2,5-chalcogeno­diazole cocrystals studied by solid-state NMR spectroscopy (Nag *et al.*, 2022 ▸). All figures are reproduced from the cited references with permission.

Three articles focus solely on chalcogen bonds (Fig. 4 ▸) and reflect the growing importance and inter­est in this class of inter­actions, alongside the more extensively studied halo­gen bond. Huber and co-workers report the synthesis and crystal structures of a set of four 1,3-bis­(benzimidazolium­yl)benzene-based com­pounds designed for two-point binding of suitable Lewis bases *via* chalcogen bonds (Steinke *et al.*, 2023 ▸). This class of com­pounds and related triazolium analogues have been used successfully as Lewis acid catalysts (with chalcogens Ch = S, Se or Te) in several benchmark reactions (Wonner *et al.*, 2017 ▸, 2019*a* ▸,*b* ▸). The crystal structures reported are of the methyl­imidazolium analogues of the more soluble octylimidazolium catalysts, which proved too difficult to crystallize but exhibited some unexplained trends in catalytic behaviour as a function of chalcogen choice. The current study demonstrates in most cases two-point chalcogen-bond binding of the CF_3_SO_3_^−^ counter-ion involving one or two anion oxygen sites and exhibits single chalcogen bonds to the anion in others. These inter­actions are supported by other inter­actions, such as anion–π inter­actions, in some cases. The crystallographic studies are com­plemented by DFT calculations of mol­ecular electrostatic potentials. White and co-workers report a study of the pyridin-3-yl derivative of the selenium-containing drug ebselen, **1** (Xu *et al.*, 2023 ▸), and its methyl­pyridinium iodide salt (**1**-Me^+^I^−^) and tosyl­ate salt (**1**-Me^+^CH_3_C_6_H_4_SO_3_^−^·3H_2_O). The crystal structure of the parent drug (Dupont *et al.*, 1990 ▸) and a subsequent charge–density study (Thomas *et al.*, 2015 ▸) revealed a short and strong C—Se⋯O=C chalcogen bond [Se⋯O = 2.522 (1) Å] accom­panied by an IR stretching fre­quency shift [Δν(CO) ≃ 71 cm^−1^]. The newly reported structures contain chalcogen bonds: C—Se⋯N and C—Se⋯O=C in **1**, C—Se⋯O=C in **1**-Me^+^CH_3_C_6_H_4_SO_3_^−^·3H_2_O and a very strong inter­action that approximates the formation of an Se—I bond as a hypervalent Se. In **1**, the C—Se⋯N inter­action is much shorter than C—Se⋯O=C. In each structure, the chalcogen bonding is stronger *trans* to the N—Se covalent bond than *trans* to the C—Se covalent bond and leads to a significant lengthening of this bond, consistent with charge transfer to the N—Se σ* orbital and correlating with the strength of the chalcogen bond. An experimental charge–density study of **1** enables a more detailed analysis of the chalcogen bonding within the QTAIM (quantum theory of atoms in mol­ecules) framework (Bader, 1991 ▸). This analysis indicates that the C—Se⋯N chalcogen bond exhibits significant electron sharing (albeit with a bond critical point, BCP, consistent with a closed-shell inter­action), whereas the weaker C—Se⋯O=C chalcogen bond exhibits characteristics of a largely electrostatic inter­action. Bryce and co-workers have used single-crystal and powder X-ray diffraction alongside ^77^Se/^125^Te magic-angle spinning solid-state NMR spectroscopy in characterizing chalcogen bonding in cocrystals of 3,4-di­cyano-1,2,5-chalcogeno­diazo­les (Ch = Se and Te) with hydro­quinone or chloride as the chalcogen-bond acceptor (Nag *et al.*, 2022 ▸). The three crystal structures reported each show chalcogen-bond formation *trans* to both N—Ch covalent bonds [N—Se⋯N, N—Se⋯O, N—Te⋯Cl and N—Te⋯π(phen­yl)]. All are relatively strong (*R*_SeN_ = 0.86, *R*_SeO_ = 0.88 and *R*_TeCl_ = 0.69–0.80; N—Ch⋯*A* = 168–175°). NMR data demonstrate the sensitivity of ^125^Te chemical shift values to N—Te⋯*A* chalcogen bonds and are consistent with earlier studies of cocrystals of di­cyano-1,2,5-telluro­diazole with a variety of chalcogen-bond acceptors (*A*) (Kumar *et al.*, 2020 ▸). The 3,4-di­cyano-1,2,5-seleno­diazole–hydro­quinone cocrystal shows ^77^Se chemical shift tensor values indicative of retention of self-com­plementary N—Se⋯N chalcogen bonds.

## Conclusions

In this review our aim has been to summarize the current status of halo­gen bonds and other σ-hole inter­actions involving *p*-block elements in Lewis acidic roles, notably chalcogen bonds, pnictogen bonds and tetrel bonds. Our approach has been to tabulate and briefly discuss and classify review articles written mostly since 2013 that cover this topic. The present review also serves as an overview and introduction to the special issue in this journal com­prising 11 articles and entitled ‘*Halogen, chalcogen, pnictogen and tetrel bonds: structural chemistry and beyond*,’ which presents a snapshot of some of the current research activities in the field.

## Note (see *Introduction*)

The two early 19th century articles by Colin, entitled ‘*Sur Quelques Combinaisons de l’Iode*’ (Colin, 1814) and ‘*Memoire sur les Combinaisons de l’Iode avec Substances Vegetales et Animales*’ (Colin & Gaultier de Claubry, 1814), which are frequently cited as the earliest examples of halo­gen bonding, are sometimes incorrectly cited in the literature. The principal author, Jean-Jacques Colin, is listed as M. Colin in the former article, but this is an abbreviation for Monsieur Colin. In the latter article, the authors are listed as MM. Colin et H. Gaultier de Claubry, but this is an abbreviation for Messieurs Colin and H. Gaultier de Claubry.

## Figures and Tables

**Table 1:** List of reviews on halo­gen bonding since 2013

Title	Reference
**General**	
Metal Centers as Nucleophiles: Oxymoron of Halogen Bond-Involving Crystal Engineering	Ivanov *et al.* (2022 ▸)
Words in supra­molecular chemistry: the ineffable advances of polyiodide chemistry	Savastano (2021 ▸)
The Halogen Bond	Cavallo *et al.* (2016 ▸)
Halogen Bonding in Hypervalent Iodine Compounds	Catalano *et al.* (2016 ▸)
Halogen Bonding in Supra­molecular Chemistry	Gilday *et al.* (2015 ▸)
Halogen bonding I: Impact on materials chemistry and life sciences	Cavallo *et al.* (2015 ▸)
Halogen bonding II: Impact on materials chemistry and life sciences	Kolář *et al.* (2015 ▸)
	
**Theoretical perspectives**	
Application of Halogen Bonding to Organocatalysis: A Theoretical Perspective	Yang & Wong (2020 ▸)
Charge Displacement Analysis – A Tool to Theoretically Characterize the Charge Transfer Contribution of Halogen Bonds	Ciancaleoni *et al.* (2020 ▸)
Modern level for properties prediction of iodine-containing organic com­pounds: the halo­gen bonds formed by iodine	Bartashevich *et al.* (2017 ▸)
Computer Modelling of Halogen Bonds and Other σ-Hole Inter­actions	Kolář & Hobza (2016 ▸)
The many faces of halo­gen bonding: a review of theoretical models and methods	Wolters *et al.* (2014 ▸)
Inter­play between non-covalent inter­actions in com­plexes and crystals with halo­gen bonds	Bartashevich & Tsirelson (2014 ▸)
	
**Halogen bonds in solids**	
Recent Progress of Noncovalent Inter­action-Driven Self-Assembly of Photonic Organic Micro/Nanostructures	Ma *et al.* (2022 ▸)
Crystal engineering strategies towards halo­gen-bonded metal–organic multi-com­ponent solids: salts, cocrystals and salt cocrystals	Nemec *et al.* (2021 ▸)
Characterizing Supra­molecular Architectures in Crystals Featuring I⋯Br Halogen Bonding: Persistence of *X*⋯*X*′ Secondary-Bonding in Their Congeners	Tiekink (2021*a* ▸)
Halogen bonding in the co-crystallization of potentially ditopic di­iodo­tetra­fluoro­benzene: a powerful tool for constructing multicom­ponent supra­molecular assemblies	Ding *et al.* (2020 ▸)
Halogen Bonding: A Halogen-Centered Noncovalent Inter­action Yet to Be Understood	Varadwaj *et al.* (2019 ▸)
From Mol­ecules to Inter­actions to Crystal Engineering: Mechanical Properties of Organic Solids	Saha *et al.* (2018 ▸)
Mol­ecular Recognition with Resorcin[4]arene Cavitands: Switching, Halogen-Bonded Capsules, and Enanti­oselective Complexation	Gropp *et al.* (2018 ▸)
Co-crystallization of 1,3,5-tri­fluoro-2,4,6-tri­iodo­benzene (1,3,5-TFTIB) with a variety of Lewis bases through halo­gen-bonding inter­actions	Ding *et al.* (2017 ▸)
Crystallography of encapsulated mol­ecules	Rissanen (2017 ▸)
Halogen bonding: A powerful, emerging tool for constructing high-dimensional metal-containing supra­molecular networks	Li *et al.* (2016 ▸)
Halogen Bonds in Crystal Engineering: Like Hydrogen Bonds yet Different	Mukherjee *et al.* (2014 ▸)
Alternative Motifs for Halogen Bonding	Troff *et al.* (2013 ▸)
The Halogen Bond in the Design of Functional Supra­molecular Materials: Recent Advances	Priimagi *et al.* (2013 ▸)
	
**Halogen bonds on surfaces**	
Halogen Bonds Fabricate two-dimensional Mol­ecular Self-Assembled Nanostructures by Scanning Tunneling Microscopy	Wang *et al.* (2020*b* ▸)
Halogen Bonding in Two-Dimensional Crystal Engineering	Teyssandier *et al.* (2020 ▸)
	
**Halogen bonds in solution and gas phase**	
Halogen bonding motifs for anion recognition	Pancholi & Beer (2020 ▸)
Halogen bonds of halonium ions	Turunen & Erdélyi (2020 ▸)
Halogen bonding in solution: NMR spectroscopic approaches	von der Heiden *et al.* (2020 ▸)
The Hydrogen Bond, the Halogen Bond and Rotational Spectroscopy: A Personal Retrospective	Legon (2020 ▸)
Helical Anion Foldamers in Solution	John *et al.* (2020 ▸)
Halogen Bonding in Solution: Anion Recognition, Templated Self-Assembly, and Organocatalysis	Tepper & Schubert (2018 ▸)
Characterization of Halogen Bonded Adducts in Solution by Advanced NMR Techniques	Ciancaleoni (2017 ▸)
Anion Recognition Strategies Based on Combined Noncovalent Inter­actions	Molina *et al.* (2017 ▸)
Halogen bond symmetry: the N—*X*—N bond	Hakkert & Erdélyi (2015 ▸)
Advances in Anion Supra­molecular Chemistry: From Recognition to Chemical Applications	Evans & Beer (2014 ▸)
Halogen bonding in solution: thermodynamics and applications	Beale *et al.* (2013 ▸)
	
**Applications: materials/synthesis**	
Halogen bonding regulated functional nanomaterials	Zheng *et al.* (2021 ▸)
Non-covalent inter­actions (NCIs) in π-conjugated functional materials: advances and perspectives	Haque *et al.* (2023 ▸)
Stereoselective Processes Based on σ-Hole Inter­actions	Peluso & Mamane (2022 ▸)
Halogen Bonding in Perovskite Solar Cells: A New Tool for Improving Solar Energy Conversion	Metrangolo *et al.* (2022 ▸)
Halogen bonding in polymer science: towards new smart materials	Kampes *et al.* (2021 ▸)
Bridging the Void: Halogen Bonding and Aromatic Inter­actions to Program Luminescence and Electronic Properties of π-Conjugated Materials in the Solid State	Sharber *et al.* (2021 ▸)
Halogen bond-induced electrophilic aromatic halo­genations	Lorpaiboon & Bovonsombat (2021 ▸)
An up-to-date review on halo­gen-bonded liquid crystals	Devadiga & Ahipa (2021 ▸)
Halogen bonding in room-temperature phospho­rescent materials	Wang *et al.* (2020*a* ▸)
Organic halo­gen-bonded co-crystals for optoelectronic applications	Chen *et al.* (2020 ▸)
Supra­molecular Halogen Bonds in Asymmetric Catalysis	Kaasik & Kanger (2020 ▸)
Recent Advances in Halogen Bond-assisted Organic Synthesis	Yamada & Konno (2020 ▸)
Enhanced Room-Temperature Phospho­rescence through Inter­molecular Halogen/Hydrogen Bonding	Xiao & Fu (2019 ▸)
Halogen Bonding beyond Crystals in Materials Science	Saccone & Catalano, (2019 ▸)
Electrochemical activation of halo­gen bonding	Fave & Schöllhorn (2019 ▸)
Halogen-Bonded Cocrystals as Optical Materials: Next-Generation Control over Light-Matter Inter­actions	Christopherson *et al.* (2018 ▸)
Halogen bonding in polymer science: from crystal engineering to functional supra­molecular polymers and materials	Berger *et al.* (2015 ▸)
Halogen bonding at work: recent applications in synthetic chemistry and materials science.	Meyer & Dubois (2013 ▸)
	
**Biomolecules**	
Noncovalent inter­actions in proteins and nucleic acids: beyond hydro­gen bonding and π-stacking	Jena *et al.* (2022 ▸)
A Halogen Bonding Perspective on Iodo­thyronine Deiodinase Activity	Marsan & Bayse (2020 ▸)
Halogen Bonding in Biomimetic Deiodination of Thyroid Hormones and their Metabolites and Dehalo­genation of Halogenated Nucleosides	Mondal *et al.* (2020*b* ▸)
Halogen Bonding in the Mol­ecular Recognition of Thyroid Hormones and Their Metabolites by Transport Proteins and Thyroid Hormone Receptors	Mondal *et al.* (2020*a* ▸)
Hydrogen Bond Enhanced Halogen Bonds: A Synergistic Inter­action in Chemistry and Biochemistry	Riel *et al.* (2019 ▸)
Directing Traffic: Halogen-Bond-Mediated Membrane Transport	Govindaraj *et al.* (2019 ▸)
Halogen bonding in halocarbon–protein com­plexes and com­putational tools for rational drug design	Costa *et al.* (2019 ▸)
Looking Back, Looking Forward at Halogen Bonding in Drug Discovery	Mendez *et al.* (2017 ▸)
Halogen bonds involved in binding of halo­genated ligands by protein kinases	Poznański *et al.* (2016 ▸)
Mol­ecular Recognition in Chemical and Biological Systems	Persch *et al.* (2015 ▸)
Synthetic Ion Transporters that Work with Anion–π Inter­actions, Halogen Bonds, and Anion–Macrodipole Inter­actions	Vargas Jentzsch *et al.* (2013 ▸)
Halogen bonding (X-bonding): A biological perspective	Scholfield *et al.* (2013 ▸)

**Table 2:** List of reviews on chalcogen bonding since 2013

Te⋯N secondary-bonding inter­actions in tellurium crystals: Supra­molecular aggregation patterns and a com­parison with their lighter congeners	Tiekink (2022 ▸)
Metal Coordination Enhances Chalcogen Bonds: CSD Survey and Theoretical Calculations	Frontera & Bauza (2022 ▸)
Chalcogen bonding in coordination chemistry	Mahmudov *et al.* (2022 ▸)
Harnessing noncovalent inter­action of chalcogen bond in organocatalysis: From the catalyst point of view	Yan *et al.* (2021 ▸)
Supra­molecular aggregation patterns featuring Se⋯N secondary-bonding inter­actions in mono-nuclear selenium com­pounds: A com­parison with their congeners	Tiekink (2021*b* ▸)
Zero-, one-, two- and three-dimensional supra­molecular architectures sustained by Se⋯O chalcogen bonding: A crystallographic survey	Tiekink (2021*c* ▸)
Participation of S and Se in hydro­gen and chalcogen bonds	Scheiner (2021 ▸)
Chalcogen bonding in materials chemistry	Ho *et al.* (2020 ▸)
Chalcogen bonding in crystalline diselenides and seleno­cyanates: From mol­ecules of pharmaceutical inter­est to conducting materials	Fourmigué & Dhaka (2020 ▸)
Chalcogen-bond driven mol­ecular recognition at work	Biot & Bonifazi (2020 ▸)
Anion recognition using chalcogen bonding	Tanii (2020 ▸)
Chalcogen Bonding: An Overview	Vogel *et al.* (2019 ▸)
Dithieno­thio­phenes at Work: Access to Mechanosensitive Fluorescent Probes, Chalcogen-Bonding Catalysis, and beyond	Strakova *et al.* (2019 ▸)
The Chalcogen Bond in Crystalline Solids: A World Parallel to Halogen Bond	Scilabra *et al.* (2019 ▸)
Secondary Forces in Protein Folding	Newberry & Raines (2019 ▸)
Adaptive responses of sterically confined intra­molecular chalcogen bonds	Selvakumar & Singh (2018 ▸)
Mol­ecular and supra­molecular chemistry of mono- and diselenium analogues of metal di­thio­carbamates	Lee *et al.* (2018 ▸)
Chalcogen bonding in synthesis, catalysis and design of materials	Mahmudov *et al.* (2017*b* ▸)

**Table 3:** List of reviews on pnictogen bonding since 2013

The Pnictogen Bond Forming Ability of Bonded Bismuth Atoms in Mol­ecular Entities in the Crystalline Phase: A Perspective	Varadwaj *et al.* (2023*b* ▸)
The Nitro­gen Bond, or the Nitro­gen-Centered Pnictogen Bond: The Covalently Bound Nitro­gen Atom in Mol­ecular Entities and Crystals as a Pnictogen Bond Donor	Varadwaj *et al.* (2022*d* ▸)
The Phospho­rus Bond, or the Phospho­rus-Centered Pnictogen Bond: The Covalently Bound Phospho­rus Atom in Mol­ecular Entities and Crystals as a Pnictogen Bond Donor	Varadwaj *et al.* (2022*e* ▸)
The Pnictogen Bond: The Covalently Bound Arsenic Atom in Mol­ecular Entities in Crystals as a Pnictogen Bond Donor	Varadwaj *et al.* (2022*b* ▸)
The Stibium Bond or the Anti­mony-Centered Pnictogen Bond: The Covalently Bound Anti­mony Atom in Mol­ecular Entities in Crystal Lattices as a Pnictogen Bond Donor	Varadwaj *et al.* (2022*c* ▸)
The Pnictogen Bond, Together with Other Non-Covalent Inter­actions, in the Rational Design of One-, Two- and Three-Dimensional Organic–Inorganic Hybrid Metal Halide Perovskite Semiconducting Materials, and Beyond	Varadwaj *et al.* (2022*a* ▸)
Pnictogen bonding in coordination chemistry	Mahmudov *et al.* (2022 ▸)
Fluorinated elements of Group 15 as pnictogen bond donor sites	Scilabra *et al.* (2017 ▸)
The pnicogen bond: Its relation to hydro­gen, halo­gen, and other noncovalent bonds	Scheiner (2013*b* ▸)

**Table 4:** List of reviews on tetrel bonding since 2013

Recent advances on the tetrel bonding inter­action in the solid state structure of lead com­plexes with hydrazine based bis-pyridine Schiff base ligands	Banerjee *et al.* (2022 ▸)
NCIPLOT and the analysis of noncovalent inter­actions using the reduced density gradient	Laplaza *et al.* (2021 ▸)
C(*sp* ^3^) atoms as tetrel bond donors: A crystallographic survey	Daolio *et al.* (2020 ▸)
Tetrel Bonding Inter­actions Involving Carbon at Work: Recent Advances in Crystal Engineering and Catalysis	Frontera (2020 ▸)
Tetrel bonding inter­actions at work: Impact on tin and lead coordination com­pounds	Bauzá *et al.* (2019 ▸)
Close contacts involving germanium and tin in crystal structures: experimental evidence of tetrel bonds	Scilabra *et al.* (2018 ▸)
Tetrel Bonding Inter­actions	Bauzá *et al.* (2016 ▸)

**Table 5:** List of reviews (since 2013) focusing on two or more σ-hole inter­actions

Title	Reference
**Halogen, chalcogen, pnictogen and tetrel bonding**	
Recognition in the Domain of Mol­ecular Chirality: From Noncovalent Inter­actions to Separation of Enanti­omers	Peluso & Chankvetadze (2022 ▸)
The Relevance of Experimental Charge Density Analysis in Unraveling Noncovalent Inter­actions in Mol­ecular Crystals	Thomas *et al.* (2022 ▸)
A Biological Take on Halogen Bonding and Other Non-Classical Non-Covalent Inter­actions	Czarny *et al.* (2021 ▸)
Indirect spin–spin coupling constants across noncovalent bonds	Jaźwiński (2021 ▸)
Noncovalent bonds through σ- and π-hole located on the same mol­ecule. Guiding principles and com­parisons	Zierkiewicz *et al.* (2021 ▸)
On the Importance of σ-Hole Inter­actions in Crystal Structures	Frontera & Bauzá (2021 ▸)
Noncovalent Inter­actions at Lanthanide Complexes	Mahmudov *et al.* (2021 ▸)
Yet another perspective on hole inter­actions	Tarannam *et al.* (2021 ▸)
Classification of so-called non-covalent inter­actions based on VSEPR model	Grabowski (2021 ▸)
Electrostatics and Polarization in σ- and π-Hole Noncovalent Inter­actions: An Overview	Politzer & Murray (2020 ▸)
The Hydrogen Bond: A Hundred Years and Counting	Scheiner (2020 ▸)
Anion recognition based on halo­gen, chalcogen, pnictogen and tetrel bonding	Taylor (2020 ▸)
Unraveling the Nature of Weak Hydrogen Bonds and Inter­molecular Inter­actions Involving Elements of Group 14–17 *via* Experimental Charge Density Analysis	Row (2020 ▸)
Unravelling the Importance of H bonds, σ-hole and π-hole-Directed Inter­molecular Inter­actions in Nature	Pramanik & Chopra (2020 ▸)
Coordination of anions by noncovalently bonded σ-hole ligands	Scheiner *et al.* (2020 ▸)
Not Only Hydrogen Bonds: Other Noncovalent Inter­actions	Alkorta *et al.* (2020 ▸)
Solid-state NMR spectroscopy for the analysis of element-based non-covalent inter­actions	Xu *et al.* (2020 ▸)
Noncovalent inter­actions in metal com­plex catalysis	Mahmudov *et al.* (2019 ▸)
Forty years of progress in the study of the hydro­gen bond	Scheiner (2019 ▸)
A Million Crystal Structures: The Whole Is Greater than the Sum of Its Parts	Taylor & Wood (2019 ▸)
The Hydrogen Bond and Beyond: Perspectives for Rotational Investigations of Non-Covalent Inter­actions	Juanes *et al.* (2019 ▸)
σ-Hole Inter­actions in Anion Recognition	Lim & Beer (2018 ▸)
Mol­ecular electrostatic potentials and noncovalent inter­actions	Murray & Politzer (2017 ▸)
Non-covalent inter­actions in the synthesis of coordination com­pounds: Recent advances	Mahmudov *et al.* (2017*a* ▸)
Computer Modeling of Halogen Bonds and Other σ-Hole Inter­actions	Kolář & Hobza (2016 ▸)
σ-Hole Bond *versus* π-Hole Bond: A Comparison Based on Halogen Bond	Wang *et al.* (2016 ▸)
The Bright Future of Unconventional σ/π-Hole Inter­actions	Bauzá *et al.* (2015 ▸)
σ-Hole bonding: A physical inter­pretation	Politzer *et al.* (2014 ▸)
	
**Halogen and chalcogen bonding**	
Halogen bonding and chalcogen bonding mediated sensing	Hein & Beer (2022 ▸)
Noncovalent inter­actions in proteins and nucleic acids: beyond hydro­gen bonding and π-stacking	Jena *et al.* (2022 ▸)
Stereoselective Processes Based on σ-Hole Inter­actions	Peluso & Mamane (2022 ▸)
Frontiers in Halogen and Chalcogen-Bond Donor Organocatalysis	Bamberger *et al.* (2019 ▸)
Novel Noncovalent Inter­actions in Catalysis: A Focus on Halogen, Chalcogen, and Anion–π-Bonding	Breugst *et al.* (2017 ▸)
Unorthodox Inter­actions at Work	Zhao *et al.* (2016 ▸)
	
**Chalcogen and pnictogen bonding**	
Chalcogen and pnictogen bonds: insights and relevance	Shukla & Chopra (2021 ▸)
On the importance of pnictogen and chalcogen bonding inter­actions in supra­molecular catalysis	Frontera & Bauza (2021 ▸)
On the Importance of σ-Hole Inter­actions in Crystal Structures	Frontera & Bauzá (2021 ▸)
The challenge of non-covalent inter­actions: Theory meets experiment for reconciling accuracy and inter­pretation	Puzzarini *et al.* (2020 ▸)
	
**Halogen, chalcogen and pnictogen bonding**	
Continuum in H-bond and Other Weak Inter­actions (*X*–*Z*⋯*Y*): Shift in *X*–*Z* Stretch from Blue Through Zero to Red	Karir & Jemmis (2020 ▸)
σ-Hole Inter­actions in Catalysis	Breugst & Koenig (2020 ▸)
Plane-Wave Density Functional Theory Meets Mol­ecular Crystals: Thermal Ellipsoids and Inter­molecular Inter­actions	Deringer *et al.* (2017 ▸)
Detailed com­parison of the pnicogen bond with chalcogen, halo­gen, and hydro­gen bonds	Scheiner (2013*a* ▸)
On the reliability of pure and hybrid DFT methods for the evaluation of halo­gen, chalcogen, and pnicogen bonds involving anionic and neutral electron donors	Bauzá *et al.* (2013 ▸)
	
**Halogen, pnictogen and tetrel bonding**	
Hydrogen bond and other Lewis acid–Lewis base inter­actions as preliminary stages of chemical reactions	Grabowski (2020*a* ▸)
	
**Halogen and tetrel bonding**	
The σ and π Holes. The Halogen and Tetrel Bondings: Their Nature, Importance and Chemical, Biological and Medicinal Implications	Montaña (2017 ▸)
	
**Chalcogen, pnictogen and tetrel bonding**	
Supra­molecular assembly based on ‘emerging’ inter­molecular inter­actions of particular inter­est to coordination chemists	Tiekink (2017 ▸)
A Survey of Supra­molecular Aggregation Based on Main Group Element–Selenium Secondary Bonding Inter­actions – A Survey of the Crystallographic Literature	Tiekink (2020 ▸)
	
**Pnictogen and tetrel bonding**	
Pnicogen and tetrel bonds – tetra­hedral Lewis acid centres	Grabowski (2019 ▸)
